# The effect of eye movement desensitization and reprocessing on the fear of hypoglycemia in type 2 diabetic patients: a randomized clinical trial

**DOI:** 10.1186/s40359-020-00450-0

**Published:** 2020-08-08

**Authors:** Mohammadreza Sheikhi, Mohamad Moradi, Saeed Shahsavary, Zainab Alimoradi, Hamid Reza Salimi

**Affiliations:** 1 Psychiatric Nursing Department, School of Nursing, Qazvin University of Medical Sciences (QUMS), Qazvin, Iran; 2 Faculty of Nursing and Midwifery, Qazvin University of Medical Science, Qazvin, Iran; 3 Health Products Safety Research Center, Qazvin University of Medical Science, Qazvin, Iran; 4 Social Determinants of Health Research Center, Research Institute for Prevention of Non-Communicable Diseases, Qazvin University of Medical Sciences, Qazvin, Iran; 5 Qazvin University of Medical Sciences, Qazvin, Iran

**Keywords:** EMDR, Fear of hypoglycemia, Patients with diabetes type 2

## Abstract

**Background:**

The fear of hypoglycemia leads to psychological symptoms in patients with diabetes type 2. In this research, the effects of EDMR on the fear of hypoglycemia in patients with diabetes type 2 were examined.

**Methods:**

A clinical trial study was carried out with participation of 72 patients who had diabetes type 2 in Velayat Hospital. The participants were randomly assigned into control and intervention groups. The intervention group received EMDR. The required information was gleaned using a questionnaire of fear of hypoglycemia, intensity of hypoglycemia, and demographics filled out before the intervention, and 1 month and 3 months after it. The data were analyzed using descriptive statistics on SPSS Version 23. For comparison of fear of hypoglycemia in intervention and control groups, repeated measure ANOVA and Cohen d test were used.

**Results:**

The mean age of the participants in the intervention group was 43.17 ± 10.55 and in the control group was 45.86 ± 13.6. In this study, without considering the potential disruptors in the incorrect model, the intervention caused a reduction of 15 points 1 month after the completion of the intervention and a reduction of 17 points 3 month after the completion of the intervention on the scale of fear of hypoglycemia; but post-correction of potential disruptors, intervention caused a reduction of 19.5 scores 1 month after the completion and a reduction of 20.3 scores 3 months after the intervention .

**Conclusions:**

The EMDR can be used as a non-pharmaceutical treatment method to treat and alleviate the fear of hypoglycemia in type 2 diabetes patients.

**Trial registration:**

Iranian Registry of Clinical Trials: IRCT20181201041813N1, 2019/11/13.

## Background

Diabet Mellitus is a chronic and complicated disease that requires permanent medical cares and strategies to attenuate the risk of recurrence of many side-effects [1]. The primary goal in the management of diabetic patients is to maintain blood glucose levels at normal or near normal ranges using oral anti-diabetic tablets and insulin therapy [2]. Extensive therapy, especially insulin therapy, can increase the incidence of hypoglycemia, which is one of the most common and unpredictable effects of insulin therapy [3]. Hypoglycemia is common in type 2 diabetic patients. According to the study by Gehlaut et al. [4], at least 49.1% of patients; and according to the study conducted by Lamounier et al. [5],61.8% of patients had experienced hypoglycemia. In some categorizations, hypoglycemia is categorized into trivial and severe categories. Severe hypoglycemia represents life-threatening conditions and the necessity of a third-party intervention [6]. Hypoglycemia is a critical and life-threatening clinical concern that might be accompanied by psychological symptoms and might lead to impairment and death in intense cases [7]. A hypoglycemia experience might lead to fear of hypoglycemia recurrence [8]. According to a study conducted by Sacan et al., on 355 insulin-treated diabetic type 2 patients, 27.7% were reported fear of hypoglycemia [9].

Fear of hypoglycemia promotes dysfunctional behaviors in order to prevent hypoglycemia such as maintaining high blood glucose levels by limiting physical activity, reducing the required insulin dose, and increasing carbohydrate intake. These maladaptive behaviors may lead to permanent hyperglycemia and increase the risk of vascular complications [10]. Studies have shown that the fear of hypoglycemia might lead to sleep disorders, and a decrease in the quality of life in type 2 diabetic patients [11, 12]. No study has been conducted to control the fear of hypoglycemia in type 2 diabetic patients, but a variety of interventions are available to control the fear of hypoglycemia in diabetic type 1 patients; and among them, continuous glucose monitoring systems, insulin pen, insulin pump, insulin bolus, and smartphone applications are notable [13]. Another group of interventions is featured with educating the patients, blood sugar level awareness practices, improving treatment motivations, improving awareness about hypoglycemia, and remote medical interventions [14, 15]. Among these treatments, group and individual cognitive behavioral treatment has drawn more attention as it has been effective in correcting cognitive misperceptions in the patient and improving the mechanisms to adapt to the fear of hypoglycemia [16, 17].

Eye Movement Desensitization and Reprocessing (EMDR) is a short-term psychotherapy for anxiety, especially in the case of traumatic events [18]. It was introduced by Francis Shapiro in 1987. This technique facilitates information processing through removing the obstacles in information processing caused by traumatic memories so that the subject’s characteristics are changed through altering memories [19]. EMDR helps the client to learn from the negative experiences of the past, desensitize present triggers that are inappropriately distressing and incorporates templates for appropriate future actions [20]. This technique has been used for different populations and problems like children [21], victims of sexual abuse [22], anxiety disorders [23], and depression; and successful findings have been reported in return [24]. Moreover, EMDR was used to alleviate the psychological symptoms caused by physical diseases and has had promising results. For example, phantom limb pain in patients with amputation [25] and chronic pain [26]. also the theory of Marver et al. in fear Acquisition and Maintenance, can be used to explain the fear of hypoglycemia. According to this theory of the creation and perpetuation of fear, fear is first created by dependent learning and response conditions, and acts as a clean stimulus to create a conditional response. Since mental and physiological conditioned responses are frightening to the individual, avoidance and avoidance behaviors divert the individual from contact with real causes and become stronger through negative reinforcement [27]. Therefore, any behavior that will help a person to escape or avoid situations that lead to a conditional response will be reinforced over time; hence, EMDR can be used to desensitize the negative experiences of these patients and replace them with pleasant memories.

Given the fact that the fear of hypoglycemia is one of the main side effects of diabetes that leads to problems in controlling blood sugar level, taking into account that health interventions have been limited to patients with diabetes type 1 so far, and taking into account the fact that EMDR, as a non-invasive and cognitive-behavioral treatment, has been very successful, the present study, as the first attempt in the world, examines the effect of EMDR on the fear of hypoglycemia in patients with diabetes type 2. The main hypothesis of this study was to reduce the fear of hypoglycemia in type 2 diabetic patients following treatment with EMDR.

## Methods

### Study design

The study was carried out as a single blind parallel clinical trial. The study population consisted of patients with diabetes type 2 visiting the Clinic of Velayat Educational Hospital, Ghazvin-Iran from 2018 to 12 to 2020–01.

### Participants

The number of people participating in this study was determined using the first-type error with 0.05 and the second-type error 0.8; and using the amount of fear of hypoglycemia in the study of Walker et al. [28], 36 patients in each group. The inclusion criteria were: having diabetes type 2, hypoglycemic experience and diagnosed with the fear of hypoglycemia, no history of mental disorders, no visual impairment and strabismus, older than 25 years, no history of seizure, and full consent to participate in the study. Patients with symptoms of low blood sugar (such as sweating, confusion, lack of awareness, tremor, irregular movement, sudden changes in behavior or mood, hunger, burning or tingling sensation around the mouth, difficulty in concentrating, headache, and pale skin) were selected and then examined by researchers for fear of hypoglycemia.

For evaluation of fear of hypoglycemia in patients, questions such as »Are you afraid of recurrence of low blood sugar?“ “Have you reduced your activities following the fear of low blood sugar?” “Does the fear of blood sugar drop result in a reduction in insulin dose or consumption of oral anti-diabetes tablets?” “Have you consumed a lot of carbohydrates for fear of low blood sugar?” “Do you fear the blood sugar drop in your sleep?” were used. The exclusion criteria were failure to continue EMDR (impaired concentration during treatment and the occurrence of conditions such as dizziness) and reluctance to continue the study.

### Sampling

**Fig. 1:**
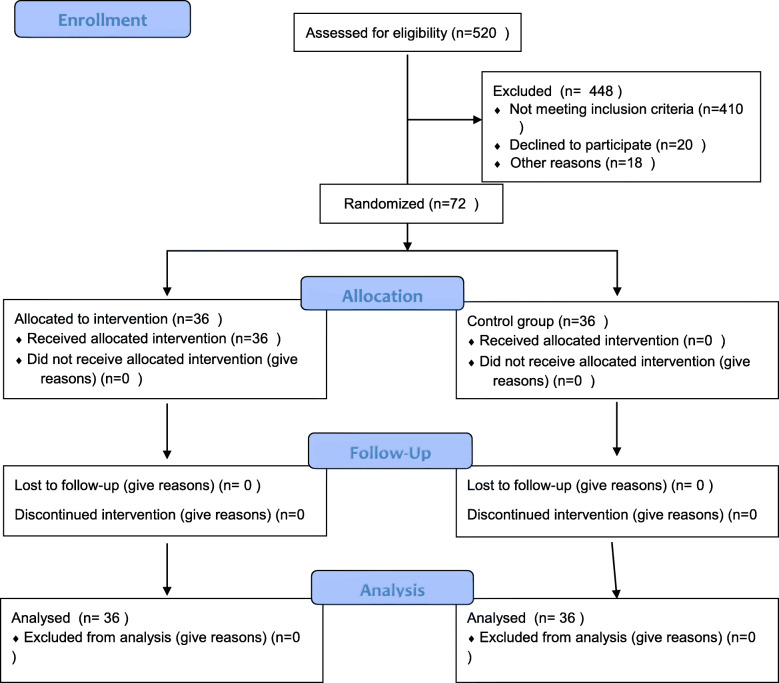
Flow diagram of participant of study

In this study, the sampling was made available. The researchers examined 520 diabetic type 2 patients and finally selected 72 patients who were eligible for the study (Fig. 1). Patients using lottery cards were divided into two groups: intervention and control. In this way, blue and red cards inside the lottery basket were presented to the patients, and each patient chose one card. In this study, red cards were assigned to the intervention group and blue cards to the control group.

### Data gathering tools

The required data were collected using a demographic questionnaire, Hypoglycemia fear survey, and Hypoglycemia severity inventory. The demographic questionnaire covered age, gender, job, education, marital status, history of diabetes, blood sugar test, awareness of insulin function, and number of hypoglycemia over the past 6 months. Fear of hypoglycemia was measured using the worry scale of fear of hypoglycemia questionnaire (HYPOGLYCEMIA FEAR SURVEY –II). This questionnaire, designed by Cox et al., included two sub-scales “worry” and “behavior.” The” worry” sub-scale is covered by 18 questions that measure fear of hypoglycemia over the past 6 months with score range from 0 to 72. The questions in this sub-scale are designed based on a five-point scale (0-- 4) and validity and reliability of the questionnaire have been supported by many studies. Momeni et al. [29] studied the validity and reliability of the tool in Iran, and obtained the Cronbach’s alpha equal to 0.96. In order to determine the severity of hypoglycemia, it was categorized based on the guideline of USA hypoglycemia association [30] in 3 groups as mild (no or low disorder in daily activities without need to treat the symptoms), moderate group (disorder in doing some activities without need for treatment), and severe group (dependence on others to treat the symptoms); and patients chose the appropriate option.

### Intervention

The intervention group received the EMDR intervention through two 45 min individual sessions in the internal ward examination room of the hospital. In each intervention session, the intervention was performed by the researcher in several stages. Each step consisted of 24 two-way cycles using finger movements [31], each cycle lasting 1 s, and the distance between the patient and the therapist during the intervention was 1 m. The intervention was performed with EMDR Standard Protocol on eight steps [32]. In phase one, a complete biography of the clients was obtained, the events that led to pathological responses in the mind were determined, and the stimuli that evoked these responses were identified. In step two, EMDR intervention and its implementation conditions were explained to patients. In step three, patients were asked to identify and focus on an image that best remembered their negative cognitions, and then they were asked to identify a positive image. In stage four, desensitization, negative cognitions were performed; in stage five, patients were asked to visualize positive cognitions in their minds. In step six, after replacing the positive cognition, patients were asked to imagine positive cognition and negative events in their minds and then review their minds and bodies and report negative experiences. In stage seven, psychological support was provided to the patients and appropriate information was given to them, and in stage eight, a reassessment was performed to ensure that all patients’ negative experiences were processed. The intervention group filled out the demographic form, hypoglycemia fear survey, and hypoglycemia severity scale before the intervention; they also filled out the hypoglycemia severity scale 1 month and 3 months after the intervention. The control group received no intervention and they only filled out the questionnaires before intervention, 1 month after and 3 months after the intervention.

### Ethical concerns

Sampling was started after securing a medical ethics license under No.: IR.QUMS.REC.1397.230 (Ethics Committee of Qazvin University of Medical Sciences) and an informed letter of consent from the participants. In addition, all the items in the Declaration of Helsinki were observed [33]. All patients were informed that the participation is voluntary and the collected information will remain confidential. All of the patients completed a written consent to participate in the study.

### Data analysis

The collected data were analyzed using descriptive statistics (frequency, percentage, mean, and SD) on SPSS (version.23) (*P* = 0.05). To compare the fear of hypoglycemia between the two groups in before intervention, after intervention and follow up, repeated measure ANOVA was used. The effect of the intervention was also measured by Cohen’s d test.

## Results

### Charachteristics of participants

All 72 selected patients in two groups continued the study and were analyzed, and none of the patients left the study. The mean age of the participants in the intervention group was 43.17 ± 10.55 and in the control group was 45.86 ± 13.6. In the intervention group, most participants (55.6%) were male; and in the control group, most participants (58.3%) were female. The mean Diabetes Duration in the intervention group was 10.5 and in the control group was 10.4 (Table 1). 77.8% of the patients in the intervention group and 66.7% of patients in the control group would check their blood sugar. Most participants in the intervention and control groups experienced moderate fear of hypoglycemia. The majority of participants in the intervention and control groups were treated with oral tablets (Table 1).

**Table 1:** Distribution of variables in intervention and control group

Group	Intervention (*N* = 36)	Control (*N* = 36)
Quantitative Variables	Mean (SD)	Mean (SD)
Age (year)	43.17 (10.55)	45.86 (13.6)
Diabetes Duration (year)	10.5 (7.22)	10.44 (8.91)
NUMBER OF Hypoglycemia	5.17 (3.41)	3.97 (3.53)
Qualitative Variables	No (%)	No (%)
Gender		
Male	20 (55.6)	15 (41.7)
Female	16 (44.4)	21 (58.3)
Marital Status		
Married	25 (69.4)	25 (69.4)
Non- Married	11 (30.6)	11 (30.6)
Educational Status		
Illiterate & Elementary	11 (30.5)	15 (41.7)
Under Diploma & Diploma	18 (50)	13 (36.1)
Academic	7 (19.5)	8 (23.2)
Job		
Unemployed	3 (8.3)	1 (2.8)
Employed	32 (88.9)	34 (94.4)
Retired	1 (2.8)	1 (2.8)
Hypoglycemia Intensity		
Mild	9 (25)	11 (30.6)
Moderate	20 (55.6)	20 (55.6)
Sever	7 (19.4)	5 (13.6)
Glucose Monitoring		
Yes	28 (77.8)	24 (66.7)
No	8 (22.2)	12 (33.3)
Type of treatment		
Insulin	13 (36.1)	8 (22.2)
Tablet	16 (44.1)	18 (50)
Mixed	7 (19.4)	10 (27.8)
Type of tablet		
Metformin	2 (12.5)	2 (11.1)
Zipmet	6 (37.5)	4 (22.2)
Metformin and Glibenclamide	5 (31.25)	7 (38.9)
Metformin and Gliclazid	3 (18.75)	5 (27.8)

### Comparing the score of fear of hypoglycemia in the control and intervention groups

**Fig. 2:**
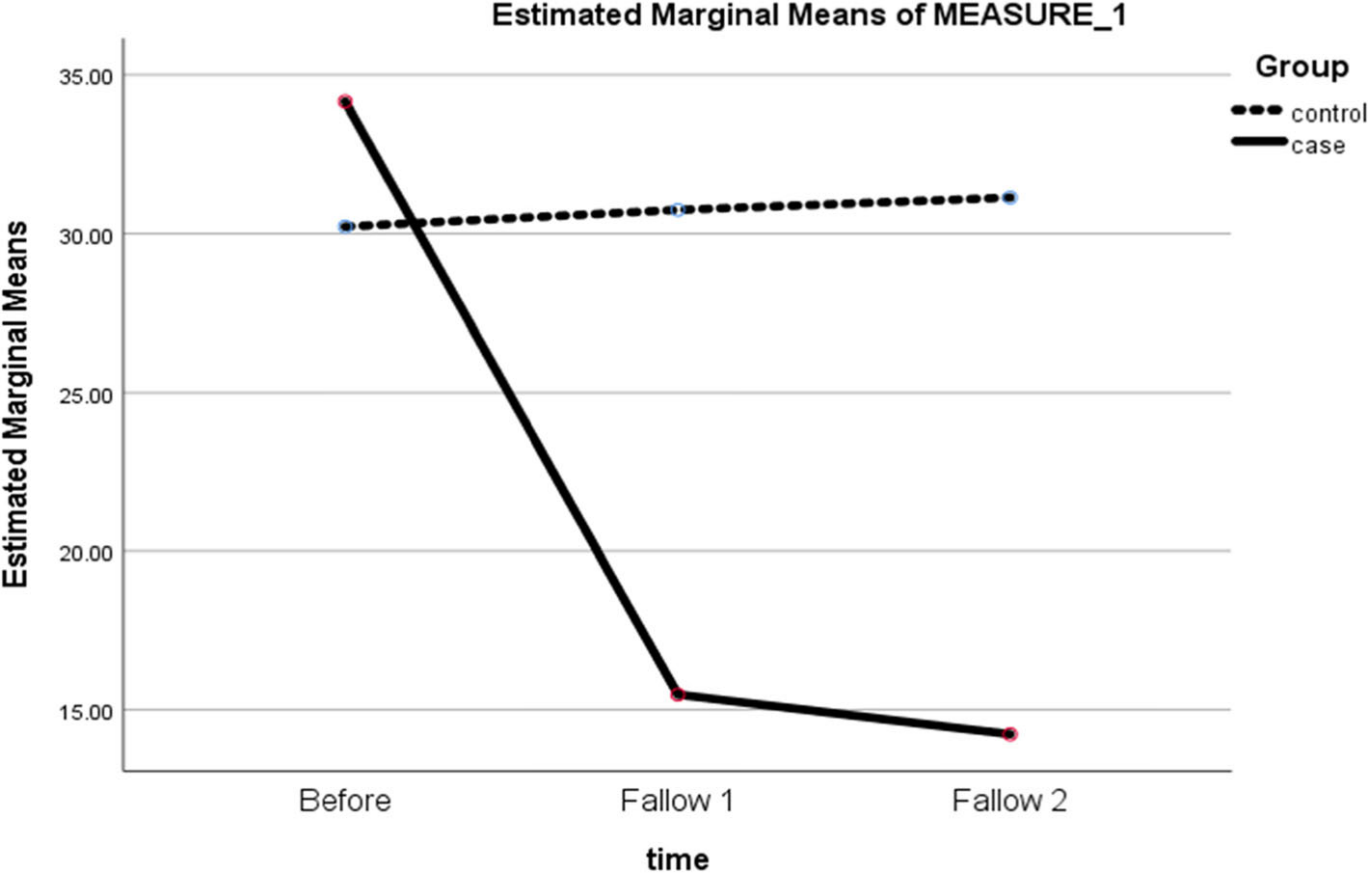
The trend of changes in the mean score of fear of hypoglycemia in study groups

The results of the study showed that without considering the potential disruptors in the incorrect model, the intervention caused a reduction of 15 points 1 month after completion of intervention and a reduction of 17 points 3 months after the completion of the intervention on the scale of fear of hypoglycemia (Fig. 2). Due to the imbalance of fear of hypoglycemia scores before the intervention, age and frequency of hypoglycemia, analysis of variance-covariance was performed. The results of the corrected model showed that the age variables and the number of hypoglycemic events did not have a significant effect on the results, but the pre-test score had a significant effect on the study results. The results of the post-correction study showed that the intervention could reduce the 19.5 score 1 month after completion and reduce the 20.3 score 3 months after the intervention on the fear of hypoglycemia score (Table 2). The size of the intervention effect based on Cohen’s d test and the partial Eta Square test in the incorrect and corrected models in terms of potential disruptors showed the size of the Large effect; so, the intervention had a significant effect on reducing the fear of hypoglycemia in patients. In addition, given that the low and high limits of the 95% D-Cohen confidence interval are within the interpretive range of the very large effect size, the result is conclusive and it seems that the volume of the present sample was sufficient to examine the effect of the intervention.

**Table 2:** The mean score of fear of hypoglycemia in the two groups before and after the intervention and follow up period

Model	Time point	Intervention *N* = 36	Comparison *N* = 36	Mean Difference 95% CI	*p*- value	Partial eta square	Cohen’s d (95% CI)
		Fear of hypoglycemic Score Mean (SD)					
Crude	Before	34.17 (10.58)	30.22 (10.5)	3.94 (−1.01;8.90)	Time: < 0.001 Group: 0.0002 Time*Group: < 0.001	0.816	−0.38 (−0.84; − 0.09)
	After	15.47 (9.85)	30.75 (10.65)	−15.28(− 10.45; − 20.1)		0.186	1.49 (0.97; 2.01)
	Fallow up	14.22 (9.4)	31.14 (10.31)	16.92 (−12.28; −21.55)		0.837	1.72 (1.18; 2.26)
Adjusted ^a^	After	13.70 (4.06)	32.53 (4.06)	−19.46 (−21.44; − 17.65)	< 0.001	0.854	4.64 (3.75; 5.53)
	Fallow up	12.55 (4.26)	32.81 (4.26)	−20.26 (−22.28; −18.24)	< 0.001	0.853	4.76 (3.85; 5.66)
Adjusted^b^	After	13.59 (4.01)	32.63 (4.01)	−19.04 (−20.97; −17.12)	< 0.001	0.853	4.75 (3.85: 5.65)
	Fallow up	12.49 (4.36)	32.87 (4.36)	−20.39 (−22.48; −-18.3)	< 0.001	0.850	4.67 (3.78; 5.57)

^a^ Adjusted for pre-intervention hypoglycemic fear score

^b^ Adjusted for pre-intervention hypoglycemic fear score + age + Number of hypoglycemic attack

## Discussion

This study was conducted for the first time with the aim of investigating the effect of EMDR treatment method on fear of hypoglycemia in type 2 diabetic patients, as a randomized clinical trial. The results of the study showed that treatment with EMDR reduces the fear of hypoglycemia in type 2 diabetic patients, and the therapeutic benefits of this method are achieved in a short time and with a significant effect size. There are no studies on the treatment of fear of hypoglycemia with EMDR method, but cognitive-behavioral interventions have been used to treat the fear of hypoglycemia. It should be noted that these interventions have been performed only in type 1 diabetic patients, and no intervention has been performed in type 2 patients, Boyle et al., in a case study, performed a cognitive-behavioral intervention in a 37-year-old woman with type 1 diabetes, who had panic attacks and fear of hypoglycemia. The implementation of this intervention led to the improvement of the idea that hypoglycemia leads to loss of control and leads to increased self-confidence in recognizing and managing blood sugar [17]. Asmebarg et al. (2009) examined the effect of cognitive behavioral intervention on type 1 diabetic patients with poor metabolic control performance. In this study, the impact of cognitive-behavioral interventions on HBA1C, self-care behaviors, and psychological factors such as fear of hypoglycemia were measured. The interventions led to a notable improvement in glycemic control, self-control, psychological factors, general welfare, perceived stress, anxiety, depression, and hypoglycemia avoidance [16]. The results of these studies are consistent with those obtained in the present study; however, these studies have been performed on type 1 diabetic patients, and the present study is on type 2 diabetic patients. In the present study, type 2 diabetic patients reported improvement in glycemic control, physical activity and physical welfare after the intervention. Patients treated with EMDR also reported that after treatment with this method, recalling past hypoglycemic events caused less stress than before the intervention.

The study also found that the effect of treatment with EMDR in management of fear of hypoglycemia in type 2 diabetic patients is permanent because after a three-month follow-up period, it was found that the fear of hypoglycemia scores in type 2 diabetic patients was reduced. In previous study of EMDR on various diseases, it was discovered that the effect of this intervention is stable over time. In a systematic review study by Tezars et al., the effect of EMDR on chronic pain was determined. The results of the study showed permanent improvement in chronic pain without any side effects [26], consistent with the results obtained in the present study; and in this study, no side effects occurred. In a study conducted by Behnam Moghadam et al. on 60 patients with myocardial infarction who had depressive symptoms, the results of a study in a 12-month follow-up period showed improvement in depressive symptoms of these patients [34]. In another study conducted by Hogberg et al. on patients with post-traumatic stress disorder, the results of the study demonstrate permanent effects of EMDR treatment in a 35-month period after the intervention [35], and these results are consistent with those in the present study. Therefore, it can be said that EMDR treatment is an effective method in controlling the fear of hypoglycemia in type 2 diabetic patients in the long time.

## Conclusion

The fear of hypoglycemia is one of the main obstacles of glycemic control in patients with diabetes type 2. The results showed that the EMDR was a successful way to treat the fear of hypoglycemia in patients with diabetes type 2. It can be considered an economic, non-invasive, and fast-rewarding method to treat the fear of hypoglycemia in patients with diabetes type 2. Further studies with longer follow-up terms are recommended to ensure the success of the treatment. Among the limitations of the study, short-term follow-up period and the self-report nature of the fear of hypoglycemia survey are notable.

## Data Availability

The datasets used and/or analysed during the current study are available from the corresponding author on reasonable request.

## Abbreviation

EMDR: Eye Movement Desensitization and Reprocessing
